# Potential value of [^68^Ga]Ga-FAPI-46 PET in patients with metastatic urothelial carcinoma: a bi-centric analysis

**DOI:** 10.1007/s00259-025-07674-5

**Published:** 2025-11-25

**Authors:** Kim M. Pabst, Sophie C. Siegmund, Adrien Holzgreve, Hans P. Schmid, Timo Bartel, Ken Herrmann, Alina T. Küper, Can Aydogdu, David Kersting, Claudia Kesch, Boris A. Hadaschik, Marcus Unterrainer, Christian G. Stief, Clemens C. Cyran, Rudolf A. Werner, Wolfgang P. Fendler, Jozefina Casuscelli, Lena M. Unterrainer

**Affiliations:** 1 Department of Nuclear Medicine, West German Cancer Centre, University Hospital Essen, Hufelandstraße 55, Essen, 45147 Germany; 2 German Cancer Consortium, Partner Site University Hospital Essen, Essen, Germany; 3 Department of Nuclear Medicine, LMU University Hospital, LMU Munich, Munich, Germany; 4 Bavarian Cancer Research Centre (BZKF), Partner Site, Munich, Munich, Germany; 5 Ahmanson Translational Theranostics Division, David Geffen School of Medicine at UCLA, Los Angeles, CA USA; 6 Department of Urology, University Hospital, LMU Munich, Munich, Germany; 7 Department of Urology, West German Cancer Centre, University Hospital Essen, Essen, Germany; 8 DIE RADIOLOGIE, Munich, Germany; 9 Department of Radiology, LMU University Hospital, LMU Munich, Munich, Germany

**Keywords:** [^68^Ga]Ga-FAPI-46 PET, 2-[^18^F]FDG-PET, Urothelial carcinoma, CeCT, Detection efficacy

## Abstract

**Purpose:**

[^68^Ga]Ga-FAPI-46 has shown promise for urothelial cancer (UC) detection. This study evaluates its diagnostic value versus contrast-enhanced CT (ceCT) and 2-[^18^F]FDG PET in the largest bi-centric cohort to date.

**Methods:**

Patients with metastatic UC undergoing [^68^Ga]Ga-FAPI-46 PET at University Hospitals Munich or Essen were retrospectively reviewed. Detection rates were compared with ceCT on a regional basis (primary, lymph nodes, visceral organs, bone). SUV_max_ and SUV_mean_ of two index lesions were recorded. In a sub-cohort, [^68^Ga]Ga-FAPI-46 and 2-[^18^F]FDG PET were compared on a lesion basis. Clinical follow-up and/or histopathology served as reference.

**Results:**

Thirty-four patients underwent [^68^Ga]Ga-FAPI-46 PET/CT and ceCT, including 10 (29%) with additional 2-[^18^F]FDG PET/CT. Across 98 lesions (*n* = 65 regions), [^68^Ga]Ga-FAPI-46 PET detected *n* = 96 (98%) and ceCT *n* = 88 (90%), with mismatch findings in eight lymph nodes (PET positive/ceCT negative) and two visceral organs (ceCT positive/PET negative). In the subgroup comparison, 78 lesions were detected in total ([^68^Ga]Ga-FAPI-46: *n* = 72 (92%); 2-[^18^F]FDG: *n* = 78 (100%)). Tumour uptake was comparable (SUV_max_ [^68^Ga]Ga-FAPI-46 PET vs. 2-[^18^F]FDG: 10.2 (IQR, 1.9) vs. 8.0 (IQR, 3.3), *p* = 0.249), whereas [^68^Ga]Ga-FAPI-46 provided higher tumour-to-background ratios (Tumour-to-liver: 12.7 (IQR, 10.3) vs. 3.8 (IQR, 1.9), *p* = 0.046; tumour-to-spleen: 8.4 (IQR, 6.6) vs. 4.6 (IQR, 0.6), *p* = 0.016).

**Conclusion:**

[^68^Ga]Ga-FAPI-46 PET demonstrated higher regional detection rates than ceCT in UC patients, particularly for lymph node metastases. Compared to 2-[^18^F]FDG, it provided superior tumour-to-background contrast but detected slightly fewer lesions. [^68^Ga]Ga-FAPI-46 PET may complement established imaging in selected scenarios, although its role in routine UC staging remains investigational.

**Supplementary Information:**

The online version contains supplementary material available at 10.1007/s00259-025-07674-5.

## Introduction

Urothelial cancer (UC), the 10th most common malignancy worldwide, is associated with smoking and other risk factors such as exposure to aromatic amines and ionising radiation [1, 2]. Most UC cases represent bladder cancer (BC), while upper urinary tract UC (UTUC) accounts only for 5–10% [3]. Standard treatment for muscle-invasive BC included radical cystectomy with pelvic lymph node dissection, often after neoadjuvant chemotherapy. Accurate staging is essential, as the risk of lymph node metastases increases proportionally with advancing local tumour stage, and the number of lymph nodes involved is significantly associated with increased cancer-specific mortality [4–6].

Current guidelines recommend contrast-enhanced computed tomography (ceCT) of the thorax, abdomen, and pelvis, or a combination of abdominal/pelvic MRI with thoracic ceCT [1]. However, discrepancies between imaging- and histopathology-based staging are common, leading to postoperative upstaging in approximately 20–25% [7, 8]. 2-[^18^F]FDG positron emission tomography/computed tomography (PET/CT), though highly specific (81–100%), shows variable sensitivity for nodal staging (23–89%), limiting its clinical adoption [8, 9]. Thus, there is an unmet need for accurate imaging for initial staging, allowing the selection of the most effective treatment option and reducing the number of unnecessary therapies performed.

A promising candidate is [^68^Ga]Ga-FAPI-46, a radioligand targeting fibroblast activation protein (FAP), which is predominantly expressed by cancer-associated fibroblasts (CAFs) within the tumour microenvironment of various malignancies [10]. In UC, FAP expression correlates with tumour stage and promotes tumour invasion [11]. Previous publications have already demonstrated that [^68^Ga]Ga-FAPI-46 PET has the potential to outperform ceCT and 2-[^18^F]FDG PET in (lymph node) staging, albeit primarily shown in small cohorts [12–14].

Here, we aimed to evaluate [^68^Ga]Ga-FAPI-46 PET in the largest UC cohort to date, comparing its detection efficacy with ceCT and 2-[^18^F]FDG PET, and to assess semiquantitative uptake characteristics.

## Materials & methods

### Patients

Patients with metastatic UC who underwent [^68^Ga]Ga-FAPI-46 PET/CT between December 2020 and December 2022 at the Departments of Nuclear Medicine at LMU University Hospital Munich and University Hospital Essen were retrospectively analysed.

All patients provided written informed consent for [^68^Ga]Ga-FAPI-46 PET/CT in accordance with the German Pharmaceuticals Act § 13(2b) at both sites. In addition, patients were enrolled either in a prospective observational study at University Hospital Essen (NCT04571086) or a registry study at LMU University Hospital Munich (IRB 24–0255). Patients were referred for additional imaging by their treating urologist/oncologist.

As part of routine clinical practice, all patients also underwent ceCT and/or 2-[^18^F]FDG PET/CT, the latter exclusively at University Hospital Essen according to institutional standard.

This analysis was conducted in accordance with the Declaration of Helsinki and its subsequent amendments and approved by the respective institutional ethics committees at LMU University Hospital Munich (IRB 24–0255) and University Hospital Essen (20–9485-BO/20–9777-BO) [15].

### Radiopharmaceuticals/radiosynthesis

According to the regulations of the German Pharmaceuticals Act § 13(2b), FAPI-labelling was supervised by the applying physician. FAPI-46 was provided by SOFIE (21000 Atlantic Blvd., Ste 730, Dulles, VA 20166). The radiolabelling was conducted as described previously [12, 16].

### Image acquisition

#### [^68^Ga]Ga-FAPI-46 PET/CT

A median activity of 207.5 MBq (interquartile range (IQR), 58.5; Munich) and 110.5 MBq (IQR, 26.0; Essen) was injected intravenously. Patients at LMU Munich additionally received furosemide (Furosemid-ratiopharm 20 mg/2 mL injection solution, ratiopharm GmbH, Ulm, Germany) for radiation protection and to reduce urinary activity in the renal pelvicalyceal system, provided there were no medical contraindications [17]. No fasting was required for [^68^Ga]Ga-FAPI-46 PET protocols. PET imaging was performed using a Siemens Biograph mCT Flow or Siemens Biograph 64 (Siemens Healthineers, Erlangen, Germany) in Munich and Biograph Vision 600 (Siemens Healthineers, Erlangen, Germany) in Essen. PET scans were conducted at a median time of 60.0 min (IQR, 7.5; Munich) and 20.5 min (IQR, 22.7; Essen) after tracer injection (Munich: 2.5 min per bed position; Essen: 1,2 mm/sec (abdomen) and 0.7 mm/sec continuous-bed-motion speed). For attenuation correction, a low-dose CT was acquired. Image reconstruction was performed iteratively using the TrueX algorithm (Munich: 3 iterations, 21 subsets; Essen: 4 iterations, 5 subsets) and Gaussian post-reconstruction smoothing (Munich: 2 mm full width at half-maximum; Essen: 4 mm full width at half-maximum).

#### Contrast-enhanced CT

All patients underwent ceCT either as part of the PET/CT scan (FAPI: 8/34; FDG: 4/34) or as a separate scan (22/34). In patients with separate ceCT, the median time interval between [^68^Ga]Ga-FAPI-46 PET and ceCT was 7 days (IQR, 13; Munich) and 5 days (IQR, 12; Essen).

#### 2-[^18^F]FDG PET/CT

An additional 2-[^18^F]FDG PET/CT scan was performed in a subset of *n* = 10 patients (26.3%) at University Hospital Essen. Fasting was required for at least 4 h prior to the scan [18]. The median injected activity of 2-[^18^F]FDG was 339 MBq (IQR, 53), and the median time from injection to acquisition was 72 min (IQR, 26). PET scans were performed on a PET/CT system (Siemens Biograph mCT or Vision, Erlangen, Germany). Additionally, intravenous contrast was administered in *n* = 5 patients (50%). The PET protocol followed current guidelines [18].

### Image analysis

#### Detection efficacy

Due to the high tumour burden in the study population, a region-based comparison of [^68^Ga]Ga-FAPI-46 PET/CT and ceCT scans was performed to assess detection efficacy. Four anatomical regions were defined for assessment: primary tumour site (P), lymph nodes (LN), visceral organs (VO), and bone (B).

For each region, the two lesions demonstrating the highest [^68^Ga]Ga-FAPI-46 uptake (as measured by maximum standardised uptake value (SUV_max_)) were selected for analysis. Additionally, all scans were systematically reviewed for discordant findings, including ceCT positive but PET negative lesions, and [^68^Ga]Ga-FAPI-46 PET positive but ceCT negative lesions.

On [^68^Ga]Ga-FAPI-46 PET/CT scans, areas with increased focal uptake above the background level were considered positive, provided they were not attributable to non-malignant findings. In ceCT scans, lymph nodes >1 cm in short diameter, with features suggestive of malignancy (e.g., contrast enhancement, round shape) were classified as positive [19]. Furthermore, morphologically delineated or hyperarterialized organ lesions on ceCT were regarded as suggestive of malignancy. The standard of truth was established using follow-up imaging (ceCT or PET/CT), clinical data, and/or histopathological confirmation.

In addition to [^68^Ga]Ga-FAPI-46 PET scans, all patients at University Hospital Essen also underwent 2-[^18^F]FDG PET scans as part of routine clinical practice. The median time interval between [^68^Ga]Ga-FAPI-46 PET and 2-[^18^F]FDG PET was 1 days (IQR, 4). Due to the lower number of patients who received both imaging modalities, and the relatively small tumour burden, a lesion-based analysis was conducted to compare the two PET modalities. Therefore, each detected lesion was considered positive, irrespective of the imaging modality used. Areas with increased focal uptake above the background level were considered positive if they were not attributable to physiological findings. Clinical follow-up imaging (ceCT or PET/CT), clinical data, and/or histopathological confirmation were used as the reference standard.

[^68^Ga]Ga-FAPI-46 and 2-[^18^F]FDG PET readings were performed by one nuclear medicine physician with intermediate experience in [^68^Ga]Ga-FAPI-46 PET/CT interpretation (30–300 reads, as defined by Mei et al. [20], and with approximately five years of experience in 2-[^18^F]FDG PET/CT reporting. Ambiguous findings were reviewed and resolved in consensus with a second experienced reader.

#### Semiquantitative parameter analysis of [^68^Ga]Ga-FAPI-46 PET

Semiquantitative parameters, including maximum and mean standardised uptake values (SUV_max_ and SUV_mean_), were evaluated for the entire cohort. For each region (P, LN, VO, B), the lesion with the highest uptake was assessed to determine the SUV_max_ value. The SUV_mean_ value was determined in a lesions volume of interest (VOI) using a 50% isocontour threshold of the SUV_max_. The average SUV_mean_ of the two lesions per region (LN, VO, B) with the highest uptake, as well as the primary were selected for analysis. Furthermore, the tumour-to-liver ratio (TLR), tumour-to-spleen ratio (TSR), and tumour-to-blood pool ratio (TBPR) were calculated by dividing the respective regional uptake (SUV_max_ and SUV_mean_) by the SUV_mean_ of the liver, spleen and blood pool. Background activity was determined using spherical VOI placed in standard locations: a 3 cm VOI in the liver and spleen, and a 1 cm VOI in the blood pool (descending aorta).

#### Subgroup analysis comparing semiquantitative parameters of 2-[^18^F]FDG PET and [^68^Ga]Ga-FAPI-46 PET

A comparative analysis of the semiquantitative parameters (SUV_max_ and SUV_mean_) of [^68^Ga]Ga-FAPI-46 and 2-[^18^F]FDG PET scans was performed on a per-patient and per-region basis for both radiotracers. Due to the small number of osseous metastases, these were grouped with visceral metastases under the category distant metastases. Furthermore, TLRs, TSRs and TBPRs, derived from the above parameters, were compared.

### Statistical analysis

Data analysis was performed using Microsoft Excel (Excel 2019, Microsoft, Redmond, WA, USA), SPSS Statistics (version 27.0; IBM), and GraphPad Prism (Version 9.5.0 (730)). Continuous variables were tested for normal distribution using the Shapiro–Wilk test. As most variables were not normally distributed, all data are presented as median and IQR for consistency. The Wilcoxon test was employed to compare semiquantitative parameters between [^68^Ga]Ga-FAPI-46 and 2-[^18^F]FDG PET on a per-patient and per-region basis. A p-value < 0.05 was considered statistically significant.

## Results

### Patient characteristics

A total of 34 patients with known metastatic UC were included, comprising 9 females (26.5%) and 25 males (73.5%), with a median age of 72 years (IQR, 14) (see Table 1). As part of the subgroup analysis, 10 of 34 (29.4%) patients underwent additional 2-[^18^F]FDG PET/CT at University Hospital Essen. At the time of imaging, two patients were receiving systemic therapy (*n* = 1 immunotherapy, *n* = 1 chemotherapy). Two additional patients had undergone multiple previous lines of systemic treatment, including chemotherapy and immunotherapy, with a treatment-free interval of at least two months prior to imaging. The majority of patients, however, underwent [^68^Ga]Ga-FAPI-46 PET/CT as part of initial staging or postoperative restaging following tumour resection. Histopathological confirmation was available in 6 of 10 patients from the Essen cohort, whereas in the Munich cohort metastatic status was primarily determined by concordant findings on ceCT and follow-up imaging. The primary diagnosis of urothelial carcinoma was histopathological confirmed in all patients. Median follow-up was 6 months (IQR, 22 months).

**Table 1:** Patient characteristics

Pat. ID	Sex	Age (years)	Site	Initial Diagnosis	Primary in situ	Metastases	if VO	Current therapy
1	M	70	LMU	Bladder	no	LN, VO*	H	
2	M	76	LMU	Bladder	no	LN		
3	F	56	LMU	Bladder	yes	LN, VO, B	H	
4	M	83	LMU	Bladder	yes	LN, VO	L	
5	F	79	LMU	UTUC	no	LN, VO	L	
6	F	81	LMU	UTUC	yes	B		
7	M	60	LMU	Bladder	yes	LN		
8	M	65	LMU	Bladder	no	LN, B		
9	M	78	LMU	Bladder	no	LN, VO	L	
10	M	87	LMU	UTUC	no	LN, VO	H	
11	F	72	LMU	UTUC	no	B, VO*		
12	M	84	LMU	Bladder	yes	LN		
13	M	64	LMU	Bladder	yes	LN, VO	H, L, P	
14	M	63	LMU	UTUC	yes	LN, VO	H	
15	F	65	LMU	Bladder	yes	LN		
16	M	80	LMU	Bladder	no	LN, B		
17	M	80	LMU	UTUC	yes	LN		
18	M	81	LMU	Bladder	no	LN, VO	H	
19	M	70	LMU	Bladder	no			
20	F	66	LMU	Bladder	yes	LN, B, VO	P	
21	M	76	LMU	UTUC	yes	LN, B		
22	M	81	LMU	UTUC	yes	LN, B, VO	L	
23	M	74	LMU	UTUC	no	LN		
24	M	73	LMU	Urethra	yes	B, VO	L	
25	F	70	UK-Essen	Bladder	no			
26	M	47	UK-Essen	Bladder	no	LN, VO	AG, P	
27	M	73	UK-Essen	Bladder	no			
28	F	76	UK-Essen	Bladder	no	B		IO
29	F	65	UK-Essen	Bladder	no	LN, VO	P	
30	M	72	UK-Essen	Bladder	no			
31	M	67	UK-Essen	UTUC	no	LN		
32	M	57	UK-Essen	Bladder	no	LN		
33	M	59	UK-Essen	Bladder	no			CTx
34	M	57	UK-Essen	Bladder	yes	LN, B		

*AG* adrenal gland, *CTx* chemotherapy, *F* female, *H* hepatic, *IO* immune therapy, *L* lung, *LN* lymph node, *M* male, *B* bone, *P* peritoneal, *UTUC* Upper tract urinary cancer, *VO *visceral organ; *ceCT morphologic positive, but PET negative

### Detection efficacy

**Fig. 1:**
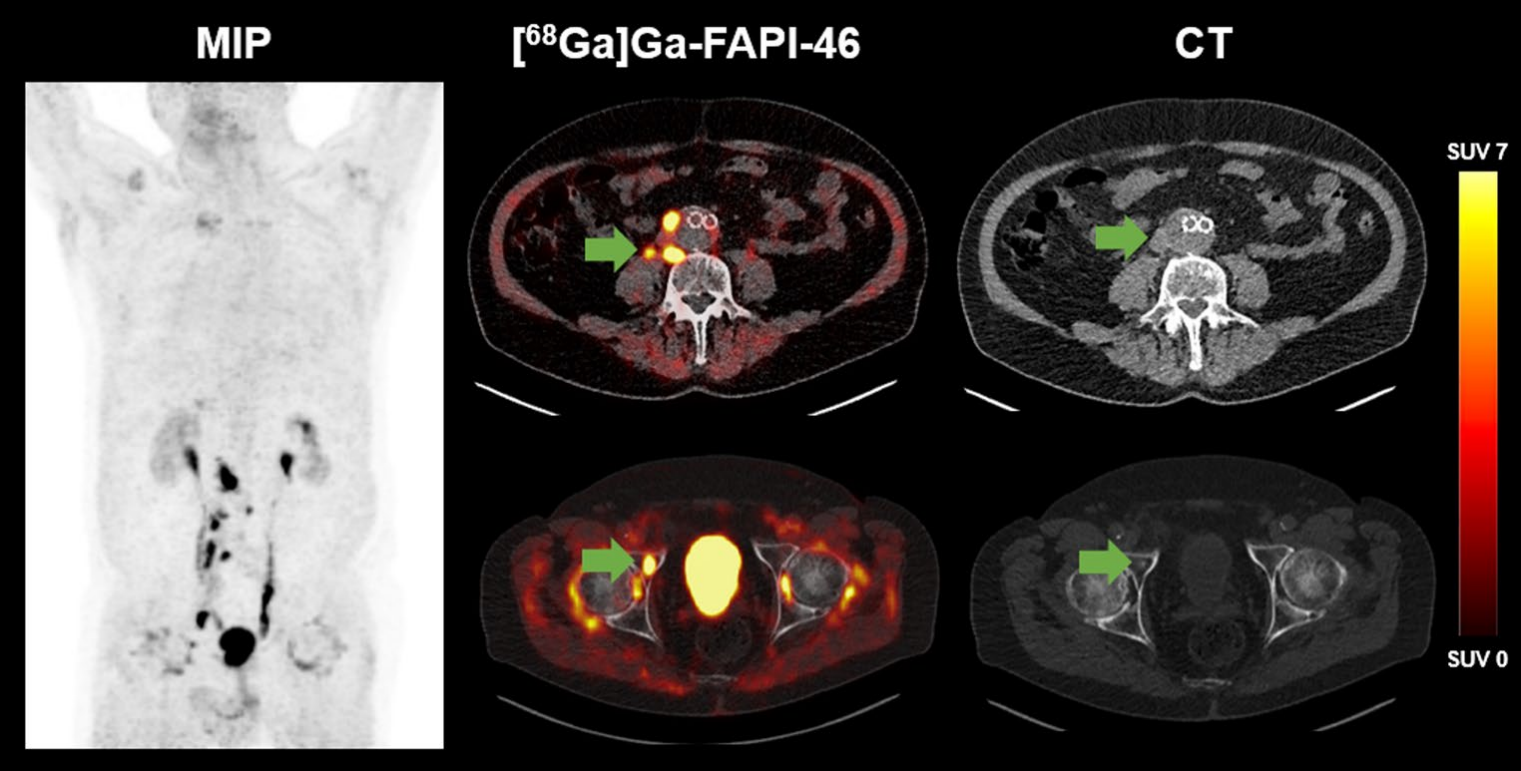
[^68^Ga]Ga-FAPI-46 PET/CT of an 81-year-old male patient with metastatic UC (LN, B). The arrows point to a paraaortic FAP-positive lymph node metastasis with a short axis diameter of 1.5 cm (SUV_max_ 45.4; upper row) and an osteoblastic lesion of the right acetabulum (SUV_max_ 18.7; lower row)

A total of 65 regions showed metastases or primary in situ. On a per-region basis, primary tumours were identified in *n* = 14 (41.2%) patients, lymph node metastases in *n* = 25 (73.5%), visceral metastases in *n* = 15 (44.1%) and osseous metastases in *n* = 11 (32.3%). Figure 1 illustrates an example of an 81-year-old patient with [^68^Ga]Ga-FAPI-46 positive lymph node and bone metastases.

**Fig. 2:**
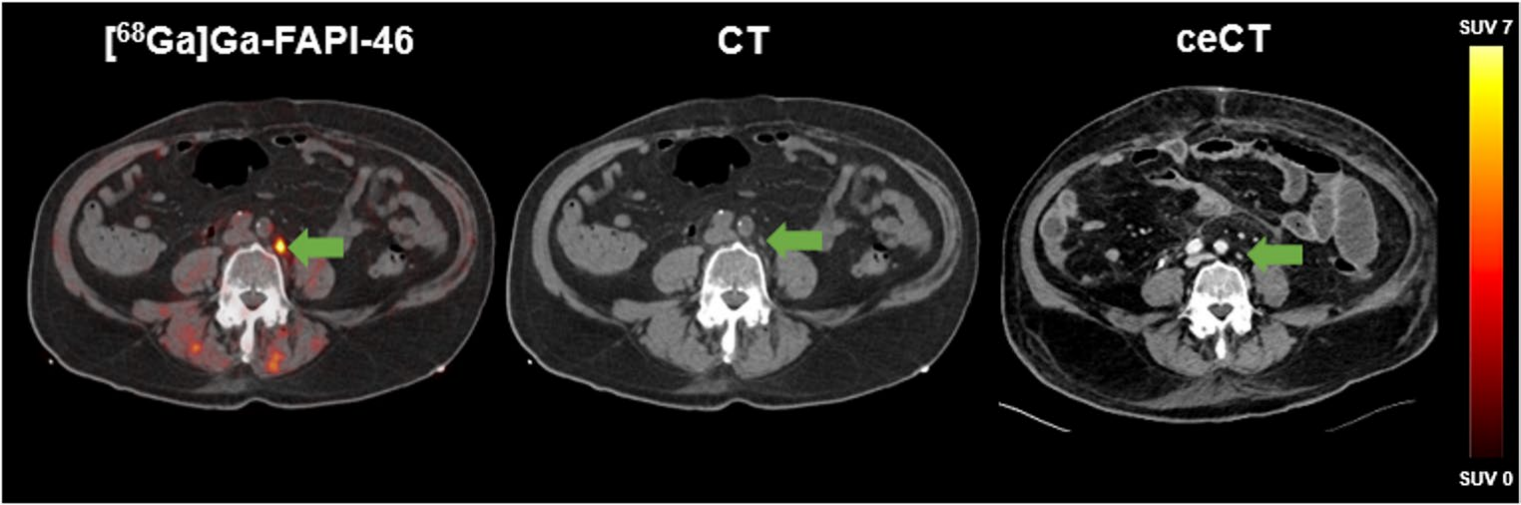
[^68^Ga]Ga-FAPI-46 PET/CT of a 84-year-old male patient with metastatic UC (LN). The arrow highlights a left sided paraaortic FAP-positive lymph node (SUV_max_ 16.6), The ceCT imaging showed no suspicious lymph node according to common CT criteria (short axis diameter: 0.8 cm)

**Fig. 3:**
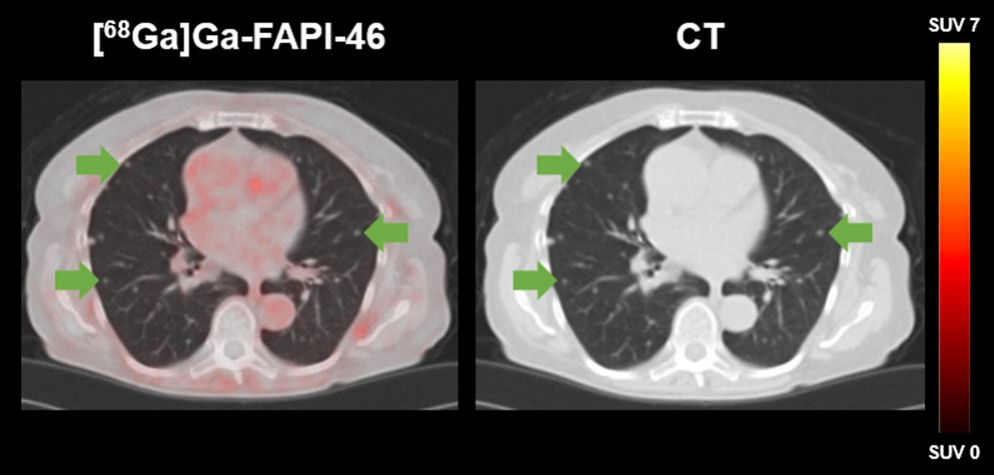
[^68^Ga]Ga-FAPI-46 PET/CT of a 72-year-old female patient with metastatic UC (B, ST, VO). The CT imaging shows multiple intrapulmonary lesions (arrows) without increased [^68^Ga]Ga-FAPI-46 uptake. Pulmonary metastases were confirmed by histopathology

The region-based analysis (two lesions per region with the highest SUV_max_) detected 98 lesions using both imaging modalities. 96/98 (98.0%) lesions were positive on [^68^Ga]Ga-FAPI-46 PET and 88/98 (89.8%) lesions on ceCT. Mismatch findings ([^68^Ga]Ga-FAPI-46 positive/ceCT negative) were observed in *n* = 8 lymph node lesions (Fig. 2). In contrast, ceCT positive/[^68^Ga]Ga-FAPI-46 negative metastases were found in *n* = 2 visceral lesions (*n* = 1 liver, *n* = 1 lung), both confirmed by histopathology and follow-up imaging (Fig. 3). Non-specific [^68^Ga]Ga-FAPI-46 uptake in joints and muscles was observed in eight patients (23.5%), most likely attributable to inflammatory processes.

**Fig. 4:**
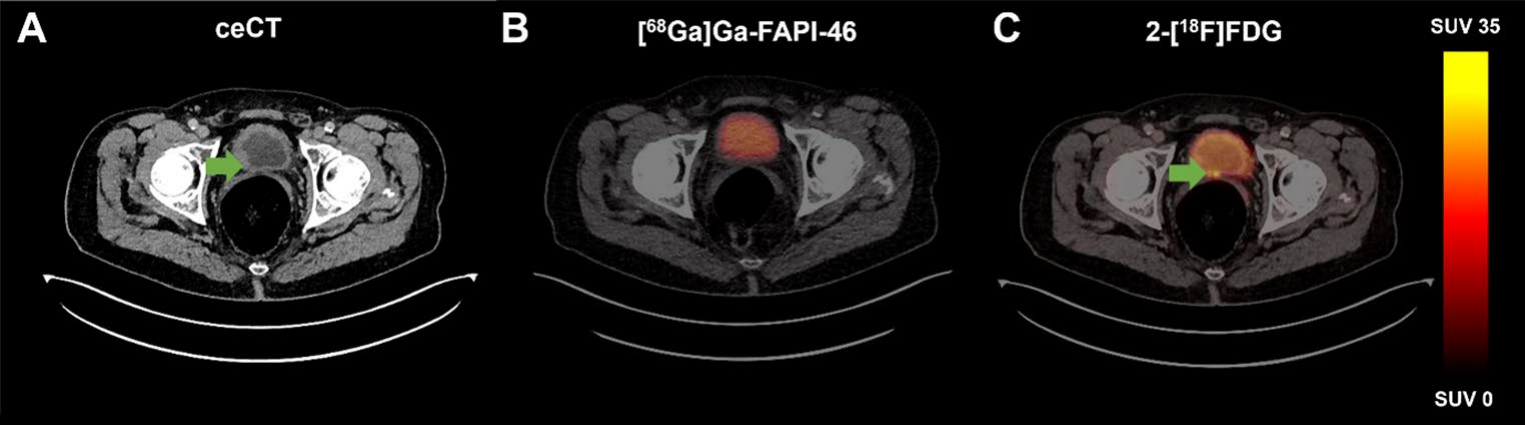
72-year-old male patient with local bladder cancer at the level of the trigonum vesicae, negative on ceCT (**A**), negative on [^68^Ga]Ga-FAPI-46 PET (**B**), and positive on 2-[^18^F]FDG PET (**C**). Histopathological confirmation of the urothelial carcinoma of the bladder was obtained within one month of imaging during a radical cystoprostatectomy with pelvic lymphadenectomy

A subgroup analysis on a per-lesion basis was performed in *n* = 10 patients who underwent [^68^Ga]Ga-FAPI-46 and 2-[^18^F]FDG PET imaging. Across both imaging modalities, *n* = 78 single lesions were detected, with *n* = 72 lesions (92.3%) on [^68^Ga]Ga-FAPI-46 PET and *n* = 78 lesions (100.0%) on 2-[^18^F]FDG PET. *N* = 3 local tumours (in one patient) and *n* = 3 lymph node metastases (in one patient) were positive on 2-[^18^F]FDG PET and negative on [^68^Ga]Ga-FAPI-46 PET. The local tumours were confirmed via histopathological workup, while the lymph node metastases were proven by a 2.5-years imaging follow-up. Figure 4 illustrates an example of a 72-year-old male patient with BC and local tumour lesions, positive on 2-[^18^F]FDG PET and negative on [^68^Ga]Ga-FAPI-46 PET.

### Semiquantitative uptake

#### Semiquantitative parameters of [^68^Ga]Ga-FAPI-46 PET

The median SUV_max_ was 11.3 and the SUV_mean_ 7.1 for all regions examined on [^68^Ga]Ga-FAPI-46 PET. Lymph node metastases demonstrated the highest uptake with a median SUV_max_/SUV_mean_ of 11.6 (IQR, 7.9)/7.6 (IQR, 5.2) followed by osseous metastases with a median SUV_max_/SUV_mean_ of 11.3 (IQR, 7.4)/7.9 (IQR, 5.4). Visceral metastases demonstrated a median SUV_max_/SUV_mean_ of 8.2 (IQR, 3.5)/5.2 (IQR, 2.1) (Table 2).

**Table 2:** Region-based [^68^Ga]Ga-FAPI-46 PET-derived SUV_max_ and SUV_mean_

	Visceral organs		Lymph nodes		Bone	
	SUV_max_	SUV_mean_	SUV_max_	SUV_mean_	SUV_max_	SUV_mean_
**Median**	8.2	5.2	11.6	7.6	11.3	7.9
**IQR**	3.5	2.1	7.9	5.2	7.4	5.4

The values presented represent the cohort-wide median SUV_max_ and SUV_mean_. Abbreviations: *IQR* interquartile range

#### Subgroup analysis comparing semiquantitative parameters of 2-[^18^F]FDG PET and [^68^Ga]Ga-FAPI-46 PET

In a subgroup of *n* = 10 patients, SUV_max_ and SUV_mean_ were compared between [^68^Ga]Ga-FAPI-46 and 2-[^18^F]FDG PET scans. No statistically significant differences were observed in either parameter on a per-patient basis (median SUV_max_ (IQR): 10.2 (1.9) for [^68^Ga]Ga-FAPI-46 vs. 8.0 (3.3) for 2-[^18^F]FDG, *p* = 0.249; median SUV_mean_ (IQR): 6.2 (1.2) for [^68^Ga]Ga-FAPI-46 vs. 4.8 (2.3) for 2-[^18^F]FDG, *p* = 0.249). Further details are provided in Supplemental Fig. 1.

In addition, a region-based analysis of SUV_max_ and SUV_mean_ of the two radiotracer was performed, stratified by lymph nodes and distant metastases (Supplemental Fig. 2). Due to the limited number of primary tumours, these were excluded from the comparative analysis. Within the analysed subgroups, there were no significant differences in SUV_max_ (lymph nodes: 10.0 (2.2) for [^68^Ga]Ga-FAPI-46 vs. 8.6 (2.9) for 2-[^18^F]FDG, *p* = 0.893; distant metastases: 10.2 (3.3) vs. 9.0 (1.2), *p* = 0.273) or SUV_mean_ (lymph nodes: 6.0 (1.5) vs. 5.4 (1.9), *p* = 0.893; distant metastases: 6.2 (2.1) vs. 5.5 (0.6), *p* = 0.273) between the two radiotracer. Supplemental Fig. 3 illustrates a representative case of a 60-year-old patient with lymph node and bone metastases showing comparable radiotracer uptake across both imaging modalities.

TBPR, TLR and TSR were compared between [^68^Ga]Ga-FAPI-46 and 2-[^18^F]FDG PET. TLR and TSR showed a significant difference in favour of [^68^Ga]Ga-FAPI-46 (TLR (IQR): 12.7 (10.3) vs. 3.8 (1.9), *p* = 0.046, TSR (IQR): 8.4 (6.6) vs. 4.6 (0.6), *p* = 0.016). Details are reported in Table 3 and Supplemental Fig. 4.

**Table 3:** Tumour-to-Background ratios

		Visceral organs		Lymph nodes		Bone	
		SUV_max_	SUV_mean_	SUV_max_	SUV_mean_	SUV_max_	SUV_mean_
**TLR**	**Median**	9.6	6.7	12.6	8.0	13.5	8.5
	**IQR**	7.3	5.4	14.9	11.7	3.4	3.7
**TBPR**	**Median**	4.4	2.8	6.1	4.0	6.0	4.2
	**IQR**	2.3	1.1	4.2	2.8	3.9	2.9
**TSR**	**Median**	9.3	5.9	12.7	8.2	12.4	8.6
	**IQR**	4.2	1.9	8.0	5.2	8.0	5.9

*TLR* Tumour-to-liver ratio, *TBPR* Tumour-to-blood pool ratio, *TSR* Tumour-to-spleen ratio, *IQR* interquartile range

## Discussion

This study confirms higher detection rates of [^68^Ga]Ga-FAPI-46 PET compared with ceCT in the largest UC cohort analysed to date, consistent with previous studies [12–14]. These findings underscore its potential as a complementary imaging modality, particularly for patients with inconclusive findings or suspected nodal involvement. In a small exploratory subgroup of ten patients, [^68^Ga]Ga-FAPI-46 PET provided superior background contrast compared with 2-[^18^F]FDG PET but detected fewer lesions (72 vs. 78).

[^68^Ga]Ga-FAPI-46 has shown promising results compared to ceCT in (lymph node) staging and 2-[^18^F]FDG PET/CT in smaller cohorts [12, 13]. In the present cohort, some PET positive regions/lesions were unsuspicious on ceCT. This is consistent with previous retrospective studies [12, 13], in which [^68^Ga]Ga-FAPI-46 PET demonstrated an advantage in identifying lymph node metastases. This finding is of particular importance, as the total number of lymph node metastases, together with the pathological tumour stage, significantly correlates with disease-specific mortality [1]. Nevertheless, two cases in our cohort demonstrated ceCT positive/[^68^Ga]Ga-FAPI-46 negative visceral metastases (in the liver and lungs), which must be considered given that accurate stage IV classification is crucial for treatment decision-making [1]. The small size of these tumour lesions and the corresponding spatial resolution may explain the absence of [^68^Ga]Ga-FAPI-46 PET uptake in these metastases. Beyond these technical factors, biological mechanisms - particularly intratumoral heterogeneity and variable stromal composition between primary and metastatic sites – may also account for the lack of tracer uptake [21]. Furthermore, the observed false-positive findings in [^68^Ga]Ga-FAPI-46 PET were likely attributable to inflammatory or fibrotic processes [22, 23].

In a lesion-based, exploratory subgroup analysis, a direct comparison between [^68^Ga]Ga-FAPI-46 and 2-[^18^F]FDG PET/CT revealed a higher lesion detection rate for 2-[^18^F]FDG (78 vs. 72 lesions), contrasting with prior findings [13]. Given the small sample size (*n* = 10) and the absence of a predefined power calculation, this head-to-head comparison should be regarded as exploratory, yet it provides valuable preliminary insights. Tumour uptake values of [^68^Ga]Ga-FAPI-46 were comparable to those of 2-[^18^F]FDG in the analysed subgroup. Previous studies have shown that FAP expression may decline as a result of treatment, potentially reducing tumoral [^68^Ga]Ga-FAPI-46 uptake at restaging. This effect may also have influenced [^68^Ga]Ga-FAPI-46 uptake in our subgroup, as two patients were receiving systemic therapy and two additional patients had undergone prior treatment lines. Although not directly observed in our data, both tracers are known to produce false-positive results in inflammatory conditions, potentially leading to misinterpretation—particularly in assessing lymph node involvement. However, [^68^Ga]Ga-FAPI-46 PET has demonstrated higher specificity in previous studies [24, 25], likely attributable to its superior tumour-to-background contrast (evoked by lower background uptake), as confirmed in our findings. Therefore, in challenging cases with equivocal imaging findings, [^68^Ga]Ga-FAPI-46 PET may offer added value for accurate disease staging. Nevertheless, given the increasing clinical use of both tracers, further prospective head-to-head studies are warranted to delineate their respective diagnostic performance and to clarify which tracer is most suitable for imaging metastatic UC, a question of considerable relevance in the field of nuclear medicine.

There are notable differences in the [^68^Ga]Ga-FAPI-46 PET/CT versus 2-[^18^F]FDG PET/CT application protocols. While patients must fast prior to 2-[^18^F]FDG PET, with a tracer uptake time of approximately 60 min [18], no fasting is required for [^68^Ga]Ga-FAPI-46 PET, and imaging is feasible as early as 10 min post-injection [26]. Therefore, [^68^Ga]Ga-FAPI-46 PET/CT might offer increased patient comfort, for example, for pain-stricken patients.

It is noteworthy that both radiotracers are excreted via the kidneys [26, 27], which consequently limits the assessment of primary tumours in the urinary tract due to reduced contrast between lesions and urinary activity. However, novel FAP-directed tracers, such as [^18^F]F-FAPI-74, are available. This fluorinated compound is primarily excreted via the biliary system [28], potentially allowing for improved delineation of primary tumours within the urinary tract.

Interestingly, [^68^Ga]Ga-FAPI-46 PET may be a valuable tool in therapeutic decision-making, as evidenced by several studies indicating that FAP-directed imaging may serve as an (early) predictor of treatment response in various tumour types, including gastric and pancreatic ductal adenocarcinoma [29, 30]. It is also conceivable that patients with localized muscle-invasive UC undergoing neoadjuvant chemotherapy may benefit from this imaging modality in the future. However, robust conclusions in this regard require prospective trials to evaluate [^68^Ga]Ga-FAPI-46 PET in this specific patient population. The data indicate that, with regard to FAP-directed radioligand therapy, further improvements in radioligand design are needed before this treatment can be considered viable for UC.

Our study comes with limitations. The retrospective design and bi-centric approach of this study resulted in a heterogeneous population. Furthermore, the imaging protocols for [^68^Ga]Ga-FAPI-46 PET/CT differed between centres. Nonetheless, both time points for imaging have been shown to be feasible and equivalent in detection efficacy [26, 31]. When comparing SUV values between centres, however, such differences in acquisition timing should be taken into account. In Munich, furosemide was administered prior to [^68^Ga]Ga-FAPI-46 PET/CT to reduce urinary tracer activity and improving lesion delineation near the bladder. However, as this study primarily focused on metastatic rather than local disease, the diagnostic impact of furosemide was likely limited. Another notable limitation is the relatively small number of patients (*n* = 10) who underwent both [^68^Ga]Ga-FAPI-46 and 2-[^18^F]FDG PET/CT, which underlines the exploratory nature of this comparative analysis and limits the generalisability of these findings.

## Conclusion

In this bi-centric retrospective study, [^68^Ga]Ga-FAPI-46 PET demonstrated higher regional detection rates than ceCT in patients with metastatic UC, particularly for lymph node metastases. In a small exploratory subgroup, 2-[^18^F]FDG PET detected slightly more lesions, while [^68^Ga]Ga-FAPI-46 PET provided superior tumour-to-background contrast. These findings support the feasibility of [^68^Ga]Ga-FAPI-46 PET as a complementary imaging tool in selected clinical scenarios, especially when conventional imaging remains inconclusive. However, its role in routine staging of UC remains investigational. Prospective, adequately powered head-to-head studies are required to define whether [^68^Ga]Ga-FAPI-46 PET can translate into clinically meaningful improvements in patient management.

## Supplementary Information

Below is the link to the electronic supplementary material.

Supplementary Material 1

Supplementary Material 2

Supplementary Material 3

Supplementary Material 4

## Data Availability

The datasets used and/or analysed during the current study are available from the corresponding author on reasonable request.
