# Supplementary figures and images for: Potential value of [68Ga]Ga-FAPI-46 PET in patients with metastatic urothelial carcinoma: a bi-centric analysis
Source: Eur J Nucl Med Mol Imaging. 2025 Nov 25;53(5):3162–71. doi: 10.1007/s00259-025-07674-5 (PMC13013314; doi:10.1007/s00259-025-07674-5)

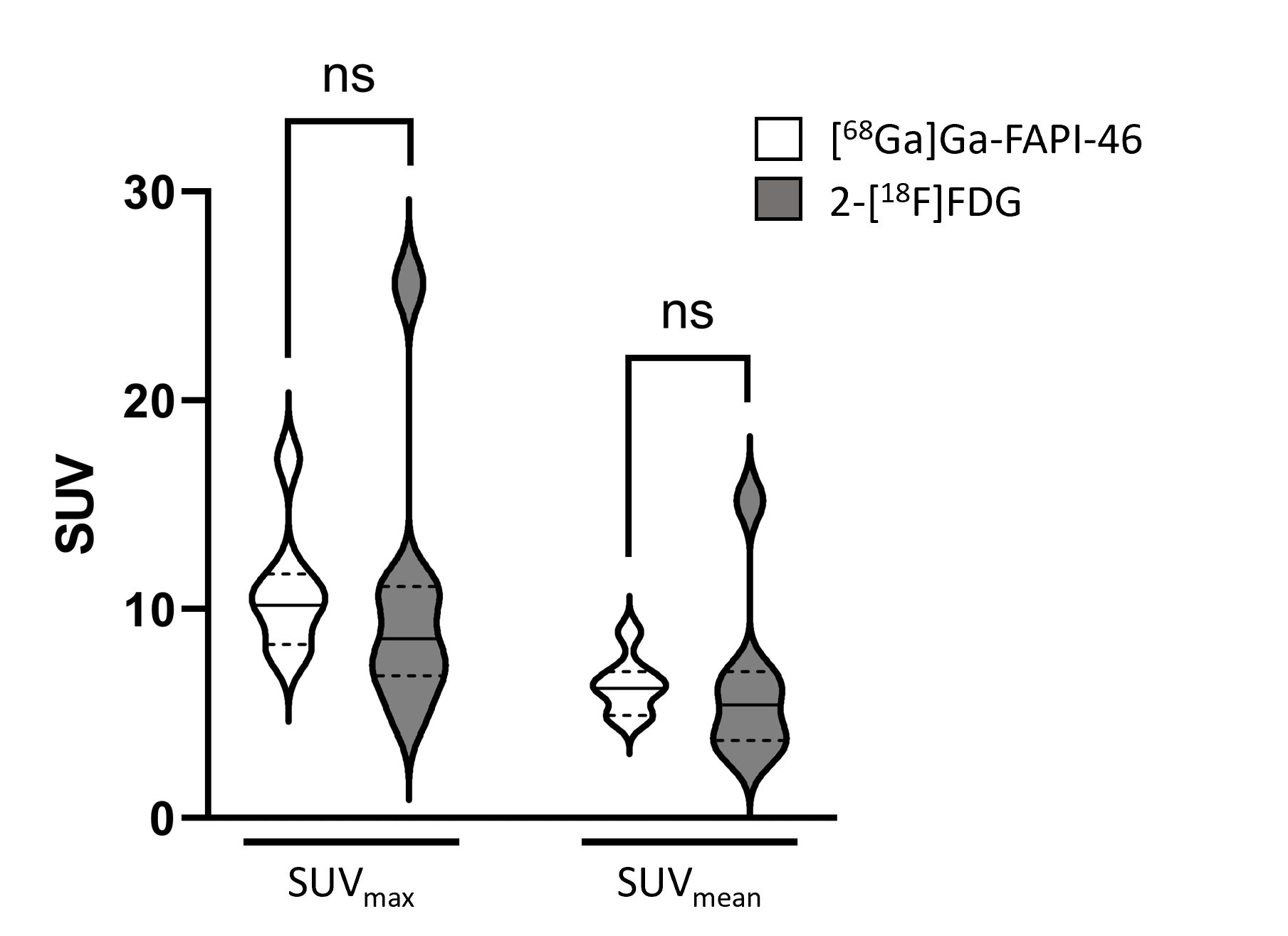

Supplement: Supplementary file 1 — Supplementary Material 1 [file 259_2025_7674_MOESM1_ESM.png]

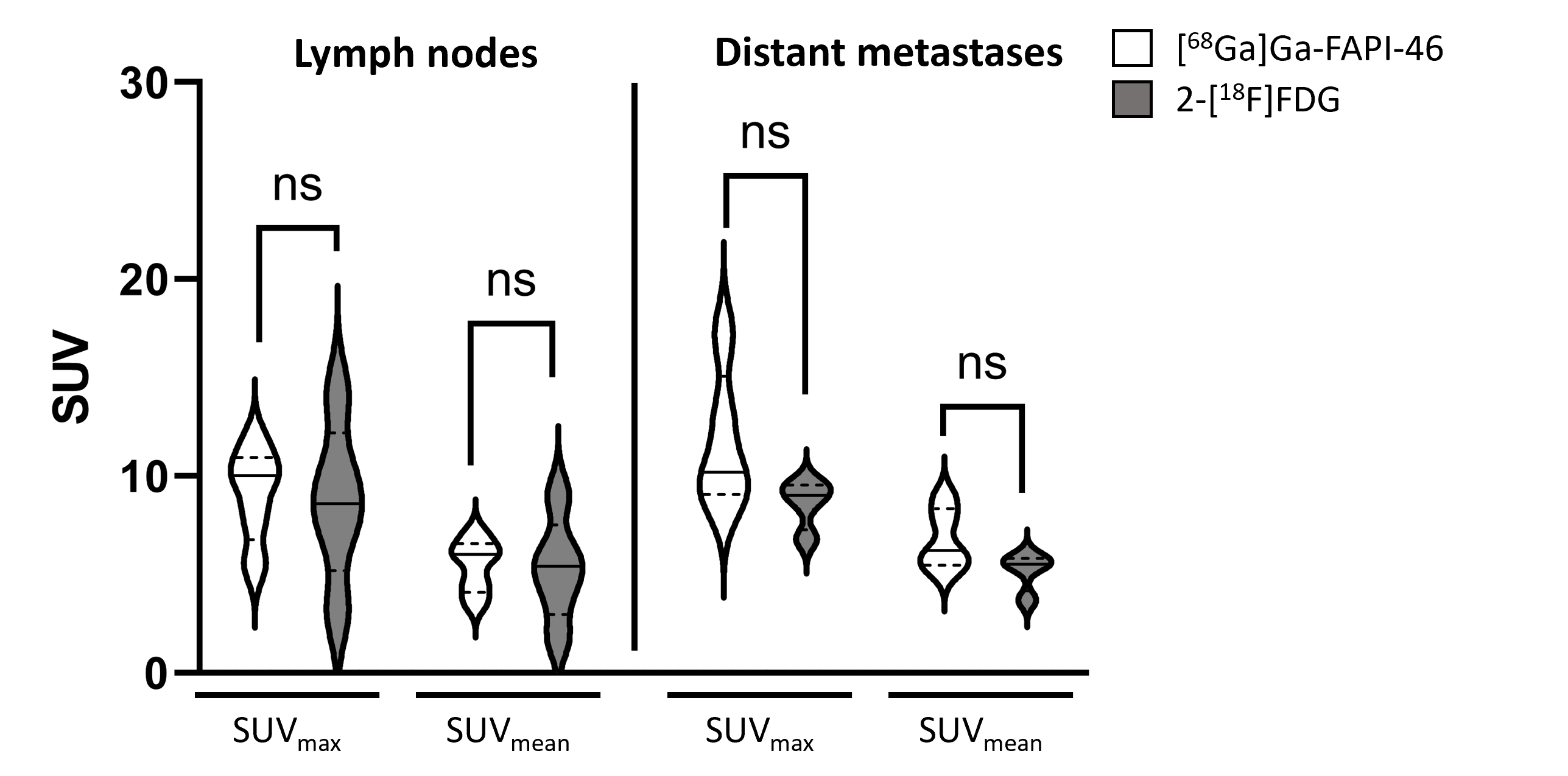

Supplement: Supplementary file 2 — Supplementary Material 2 [file 259_2025_7674_MOESM2_ESM.png]

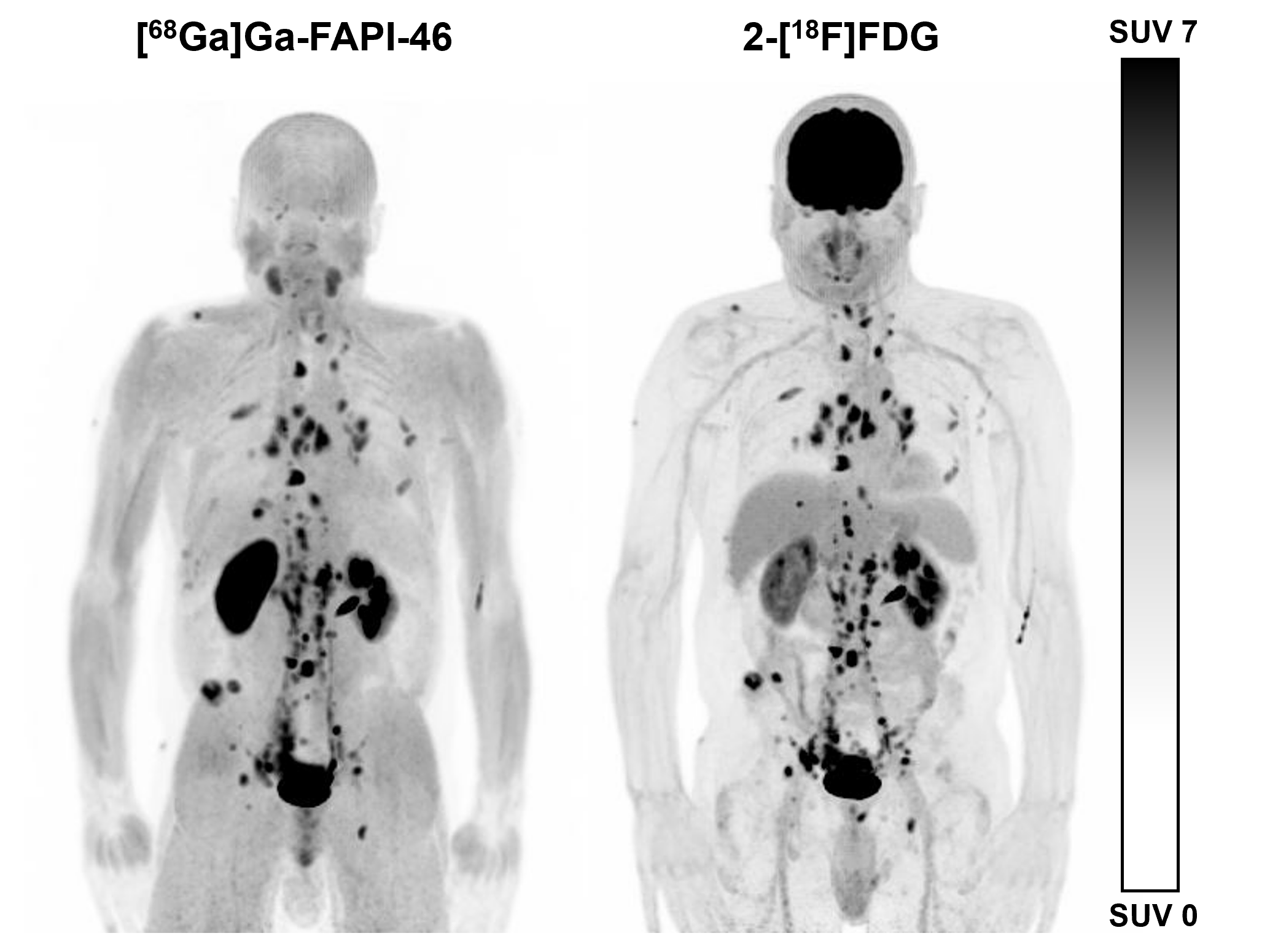

Supplement: Supplementary file 3 — Supplementary Material 3 [file 259_2025_7674_MOESM3_ESM.png]

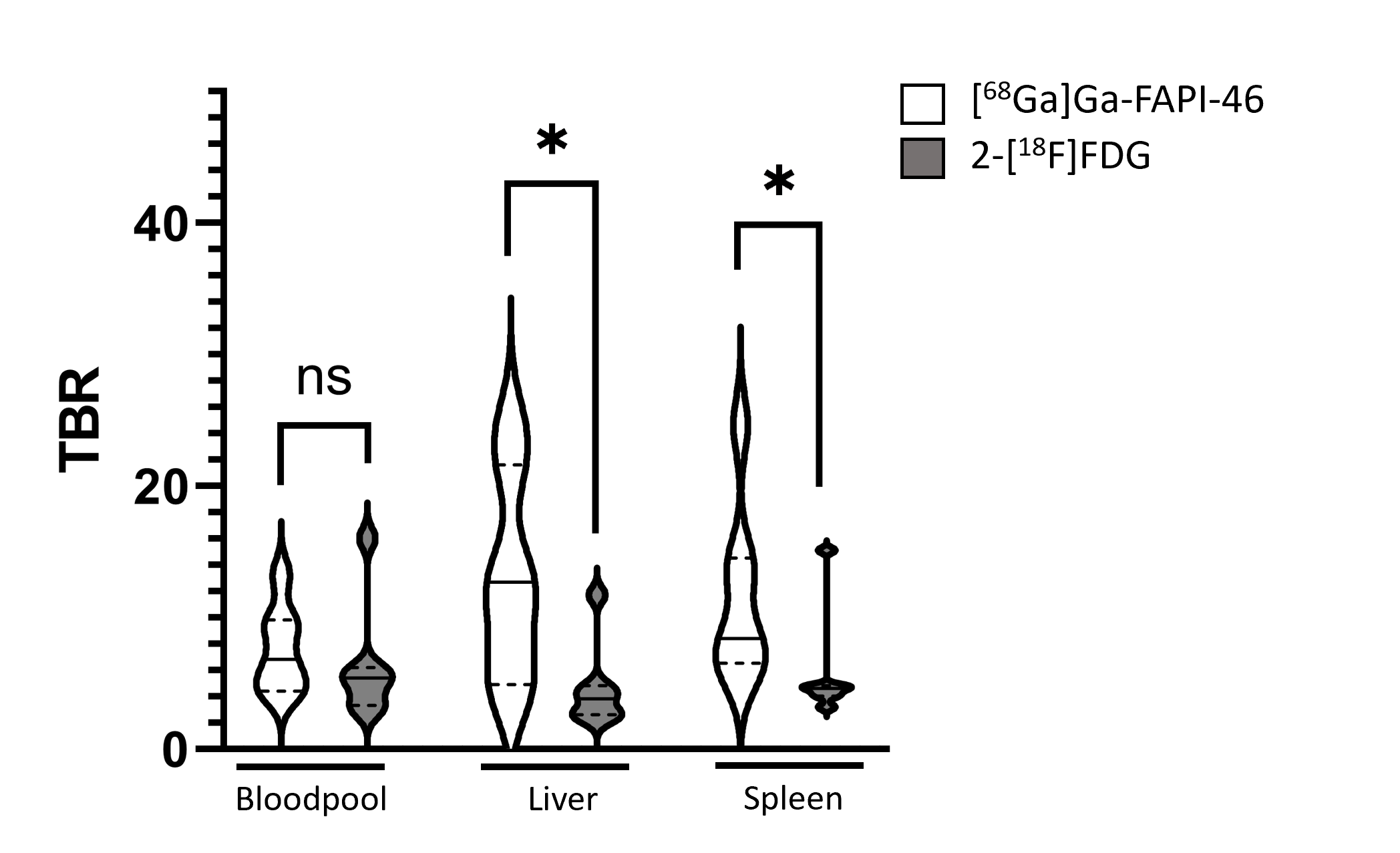

Supplement: Supplementary file 4 — Supplementary Material 4 [file 259_2025_7674_MOESM4_ESM.png]
